# Volatiles Produced by Yeasts Related to *Prunus avium* and *P. cerasus* Fruits and Their Potentials to Modulate the Behaviour of the Pest *Rhagoletis cerasi* Fruit Flies

**DOI:** 10.3390/jof8020095

**Published:** 2022-01-19

**Authors:** Raimondas Mozūraitis, Violeta Apšegaitė, Sandra Radžiutė, Dominykas Aleknavičius, Jurga Būdienė, Ramunė Stanevičienė, Laima Blažytė-Čereškienė, Elena Servienė, Vincas Būda

**Affiliations:** 1 Laboratory of Chemical and Behavioural Ecology, Institute of Ecology, Nature Research Centre, Akademijos Str. 2, LT-08412 Vilnius, Lithuania; violeta.apsegaite@gamtc.lt (V.A.); sandra.radziute@gamtc.lt (S.R.); dominykas.aleknavicius@gamtc.lt (D.A.); jurga.budiene@gamtc.lt (J.B.); laima.blazyte@gamtc.lt (L.B.-Č.); vincas.buda@gamtc.lt (V.B.); 2 Laboratory of Genetics, Institute of Botany, Nature Research Centre, Akademijos Str. 2, LT-08412 Vilnius, Lithuania; ramune.staneviciene@gamtc.lt (R.S.); elena.serviene@gamtc.lt (E.S.)

**Keywords:** yeasts, microorganisms, Diptera, Tephritidae, volatiles, attractant, repellent, electroantennography, pest management, behaviour modification

## Abstract

Yeast produced semiochemicals are increasingly used in pest management programs, however, little is known on which yeasts populate cherry fruits and no information is available on the volatiles that modify the behaviour of cherry pests including *Rhagoletis cerasi* flies. Eighty-two compounds were extracted from the headspaces of eleven yeast species associated with sweet and sour cherry fruits by solid phase micro extraction. Esters and alcohols were the most abundant volatiles released by yeasts. The multidimensional scaling analysis revealed that the odour blends emitted by yeasts were species-specific. *Pichia kudriavzevii* and *Hanseniaspora uvarum* yeasts released the most similar volatile blends while *P. kluyveri* and *Cryptococcus wieringae* yeasts produced the most different blends. Combined gas chromatographic and electroantennographic detection methods showed that 3-methybutyl acetate, 3-methylbutyl propionate, 2-methyl-1-butanol, and 3-methyl-1-butanol elicited antennal responses of both *R. cerasi* fruit fly sexes. The two-choice olfactometric tests revealed that *R. cerasi* flies preferred 3-methylbutyl propionate and 3-methyl-1-butanol but avoided 3-methybutyl acetate. Yeast-produced behaviourally active compounds indicated a potential for use in pest monitoring and control of *R. cerasi* fruit flies, an economically important pest of cherry fruits.

## 1. Introduction

Carposphere is a specific habitat populated by bacterial and fungal microorganisms including yeasts [1,2]. Berries and fruits are rich in carbohydrates and often bear the most diverse microbiome in a phyllosphere [2]. Carposphere microbiota are determined by a variety of factors such as environmental conditions, host genotype, berry developmental stage, and interactions with other organisms sharing a habitat [3,4,5,6,7]. Microorganisms associated with fruits and berries interact with insects that use these habitats for feeding and oviposition. Insects and yeasts could come into diverse relationships ranging from amensal to commensal and mutualistic [8]. Yeasts provide essential nutrients missing in sugar-rich berries that insects cannot produce while insects transfer yeasts from one substrate to another [1,9,10]. In addition to berry and fruit-related odours, yeast-produced volatiles are used by insects to acquire information about habitat quality and host choice [11,12,13,14]. The behaviour modifying effect of yeast volatiles has the potential for use in integrated pest management programs increasing the efficiency of attractive lures and serving as repellents in the push–pull pest control strategy [15].

The purpose of the study was to characterize volatile blends emitted by cultivable yeasts populating the fruit surface of sweet and sour cherries and to determine semiochemicals that modify the behaviour of *R. cerasi* fruit flies, the most important pest of cherry fruits. 

## 2. Materials and Methods

### 2.1. Yeast Sampling, Culturing, and Identification

Yeast species have been isolated from sweet cherry (*Prunus avium* L.) and sour cherry (*Prunus cerasus* L.) (Rosales: Rosaceae) fruits collected during June–July of 2018–2020 from private plantations located in the Vilnius region (GPS coordinates: 54°45′08.2″ N, 25°17′10.0″ E; 54°46′14.4″ N 25°21′04.1″ E; 54°41′19.9″ N 25°26′20.6″ E), Klaipėda region (GPS coordinates: 55°36′1.66″ N 21°36′3.8″ E; 55°34′50.6″ N 21°14′58.1″ E) and Alytus region (GPS coordinates: 54°23′43.8″ N, 23°56′18.7″ E) of Lithuania. Isolation and culturing methods have been described in detail by Stanevičienė et al. [16]. Generally, cultivable yeasts are isolated by direct rinsing of fruits with MD medium (2% dextrose, 1% (NH4)2SO4, 0.09% KH2PO4, 0.05% MgSO4, 0.023% K2HPO4, 0.01% NaCl, 0.01% CaCl_2_) or by applying fermentation-based enrichment. After cultivation on YPD-agar plates (1% yeast extract, 1% peptone, 2% dextrose, 2% agar), morphologically distinct colonies proceeded molecular analysis. For taxonomic identification of the isolates, the ITS1-5.8S rDNA-ITS2 region or D1/D2 region of 26S rDNA were PCR-amplified as described in Stanevičienė et al. [16]. To assess their taxonomic position, the resolved sequences were compared with those available in the current version of the GenBank database at the National Centre for Biotechnology Information (NCBI) (Table A1).

### 2.2. Insects

European cherry fruit flies, *Rhagoletis cerasi* (L.) (Diptera: Tephritidae), were collected as pupae from soil under sweet and sour cherry trees in April of 2019–2021 at private orchards in Vilnius (GPS coordinates: 54°45′24.0″ N 25°03′02.4″ E) and Kaunas (GPS coordinates: 54°54′13.8″ N 23°48′07.9″ E) districts, Lithuania. Reactivation of the pupae took place in a climate chamber “Fitotron” under 20–24 °C, 16L:8D (light:dark) photoperiod, and 65–75% relative humidity. Each pupa was placed in an individual 14 mL glass vial bearing wet 3 cm^2^ filter paper inside and closed by foam stoppers. The filter paper was humidified periodically to keep the humidity up inside the vial. After emergence, the adults were kept within the same vials in the room under 18–20 °C, a natural daylight photoperiod, 50–60% relative humidity, and fed on 10% sugar solution in water. The flies possessing an ovipositor were attributed to females. After sexing, each individual was kept in a separate vial under the identical conditions as described above.

### 2.3. Sampling and Analysis of Volatiles Produced by Yeasts

**Figure A1:**
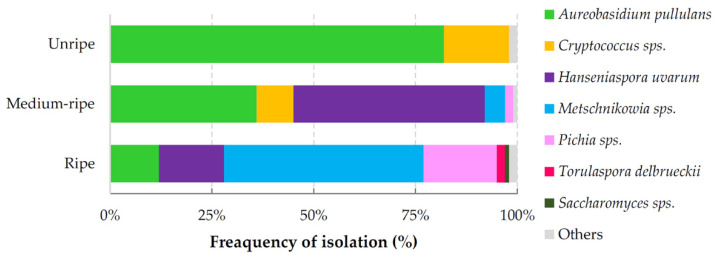
Frequency of yeast isolation among cherry samples at different ripening stages. *C. wieringae* dominated among *Cryptococcus* genus species; *M. pulcherrima* frequently occurred among *Metschnikowia* yeasts; *Pichia* genus were represented by *P. anomala*, *P. kudriavzevii*, *P. kluyveri*, *P. fermentans*, and *P. membranifaciens* yeasts; *S. paradoxus* occurred among *Saccharomyces* yeasts; others represented yeasts occurred in low frequency.

For sampling of volatile organic compounds, yeasts were selected based on isolation frequency over the different ripening stages of cherries (Figure A1). The methods have been described in detail by Lukša et al. [7]. In summary, overnight grown yeast cells (50 µL) at the concentration of about 3–5 × 10^7^ cells/mL were placed on the surface of YPD-agar medium and cultivated for two days at 25 °C. The solid-phase micro-extraction (SPME) technique was used to sample the headspace volatiles produced by yeasts. For sampling background volatiles, YPD-agar plates without yeast were used as control samples. The SPME needle was placed above the yeast culture through a small hole drilled in a Petri dish; the purified fibre coated with a polydimethylsiloxane-divinylbenzene absorbent (65 mm coating layer thickness) was exposed to the headspace for 60 min at room temperature. The volatiles collected on the fibre were desorbed for 2 min in the injection liner of a gas chromatograph (GC).

GC and mass spectrometer (MS) were used to analyse the collected volatiles. The compounds were separated by a DB-Wax column under the subsequent temperature program: isothermal at 40 °C for 1 min and afterwards gradually increased to 200 °C at a rate of 5 °C/min, then to 240 °C at a rate of 10 °C/min, and maintained isothermally for 11 min. The GC injector was run isothermally at 240 °C. Helium served as a carrier gas. The relative amount of each of the compounds was determined based on the area of the chromatographic peak. The volatile compounds were identified by comparing their mass spectra and retention indexes with those presented in a NIST version 2.0 mass spectral library and those of the available synthetic standards. C_8_–C_28_ n-alkanes were used to calculate the retention indexes of the volatiles.

### 2.4. Gas Chromatography-Electroantennogram Detection

Gas chromatographic and electroantennogram detection (EAD) techniques were applied to determine yeasts produced olfactory active volatiles to *R. cerasi* flies. A detailed description of the GC-EAD setup as well as the procedure has been published by Būda et al. [17]. Briefly, the GC was set up with a polar DB-Wax column. The injector and the detector were run at 240 °C. The oven temperature was maintained at 40 °C for 1 min; afterwards, it was raised to 240 °C at a rate of 10 °C/min, then maintained isothermally for 13 min. Hydrogen, at a flow rate of 1.5 mL/min, was used as a carrier gas. At the end of the GC column, a splitter divided an eluent into two equal parts, allowing simultaneous flame ionisation (FID) and EAD detection of the separated volatiles. A nitrogen make-up gas at 5 mL/min flow rate was added to increase FID sensitivity. The part of an eluent allocated to EAD was mixed with charcoal filtered and humidified air flowing at 0.5 m/s through a glass tube over antenna preparation. Glass capillary electrodes were used. The EAD and the FID signals were registered simultaneously, saved, and analysed. Before EAD recording, the antenna was stimulated with 1 µg of 3-methyl-1-butanol to check sensitivity. Four to seven days old flies were used in the tests. Each antenna tested was from a different fly. In total, 21 antennae of males and 18 antennae of females were used.

### 2.5. Electroantennogram Dose-Response

The same electrophysiological recording setup and the antennal preparation technique were used to record electroantennogram (EAG) dose–responses of male and female flies to the synthetic EAD active compounds: 3-methylbutyl acetate; 3-methylbutyl propionate; 2-methyl-1-butanol; and 3-methyl-1-butanol. 

The compounds were tested at the doses of 10^−5^, 10^−4^, 10^−3^, 10^−2^, and 10^−1^ mg applied in 10 µL hexane on filter paper (5 × 45 mm). The compounds were selected randomly, and five doses of each compound were tested in ascending order. A solvent blank (10 µL of hexane after evaporation) was tested as a control stimulus both at the beginning and the end of stimulation with each compound. Each EAD test was replicated 13 times, and each antenna used was from a different fly. The EAG response (R) to the EAD-active compound dose was calculated according to the formula R = RA − (RC_1_ + RC_2_)/2, where RA is the EAG response to the EAD active compound, and RC1 and RC2 are EAG responses to the first and the second control stimuli, respectively.

### 2.6. Behavioural Assay

To test the behavioural choice of the flies to the synthetic EAD active compounds versus the control, a Y-tube olfactometer [18] (25 cm main tube, 17 cm arms, 110° branching angle, the inner diameter of each arm and main tube 5 cm) was used. The olfactometer was placed in a fume cupboard. Four T8/840, Colourlux plus, 18 W tube type lamps (NARVA Lichtquellen GmbH + Co. KG, Brand-Erbisdorf, Germany) covered with a white, mat, plastic shield (65 cm length, 42 cm width) at a distance of 23 cm were placed in front of the Y tube of the olfactometer. For the fruit flies, positive phototaxis is characteristic, and the light slightly stimulated the insects to move towards the light source. Each arm of the olfactometer was connected to a glass tube that contained either the stimulus or control. A purified air delivery system CADS-4CPP (Sigma Scientific LLC, Micanopy, FL, USA) was used to push air at a rate of 0.5 L/min through each arm.

The synthetic 3-methylbutyl propionate, 2-methyl-1-butanol, and 3-methyl-1-butanol were dissolved in hexane while paraffin oil was used to dissolve 3-methylbutyl acetate and the four component mixture. Behaviour modifying effect was assessed at the few doses by dispensing the 10 µL of the solution on a filter paper strip (5 × 40 mm). The proportion of EAD active components in the mixture used in the bioassay was based on the proportion of EAD active compounds determined in the sample of *H. uvarum* yeasts. The synthetic mixture consisted of 3-methylbutyl acetate 0.55 mg, 3-methylbutyl propionate 0.05 mg, 2-methyl-1-butanol 0.012 mg, and 3-methyl-1-butanol 0.28 mg per 10 µL of paraffin oil. After 0.5 min of solvent evaporation (only applicable for samples that were dissolved in hexane and no evaporation was carried out when paraffin oil was used), the filter paper strip was placed in the glass tube connected to one arm of the olfactometer. The same size filter paper was treated either with 10 µL of hexane or with paraffin oil and was placed in the other arm serving as the control. After each test, the olfactometer was taken apart and the glassware was cleaned with hexane, soaked overnight in distilled water, and dried for 2 h in an oven, raising the temperature to 200 °C. Silicone parts of the Y-tube olfactometer were cleaned with hexane, soaked overnight in distilled water, and air-dried or replaced between the tests.

A single fly was released into the Y olfactometer at the end of the main tube. The duration within which a fly must have reached the branch point was set to 15 min. A fly was considered to have made a choice when it reached the distal end of the glass tube containing either a stimulus or a control (solvent after evaporation), irrespectively of whether the fly switched arms or not before reaching the odour source. The fly was considered as not making a choice if none of the arms was chosen within 15 min. After every five tests, the positions of the two Y-tube arms were reversed. All insects were observed individually and used in a bioassay only once. The tests were carried out at 23 ± 2 °C, 60% RH, between 10 h AM and 5 h PM local time.

### 2.7. Statistical Analysis

A nonparametric Mann–Whitney U test was applied to evaluate differences in the volatile amounts between the yeast and control samples. To assess and visualise the associations between odour blends of eleven yeast species and volatile compounds, a multidimensional scaling (MDS) analysis with a Bray–Curtis index was performed on absolute amounts expressed as areas under chromatographic peaks using R (version 4.0.2) and Rstudio (version 1.3.959), with the metaMDS function in the vegan package (version 2.5–6), and the results were visualised using ggplot2 (version 3.3.2). Prior to analysis, the data were log-transformed. Dendrogram of the odour blends was obtained by cluster analysis based on Euclidean distance using the same way transformed data as in the MDS analysis. The clustering was carried out based on the average of quantified volatile compounds from three different isolates per species. The different clusters were identified by visually evaluating the clustering. Paired *t* test was applied to compare EAG amplitudes of *R. cerasi* antennae of males versus females at each dose tested. To evaluate the choices of flies to the synthetic EAD active compounds versus the control, the total number of flies that made a choice was analysed with a χ2 test (observed vs. expected). All the analysis except MDS was performed using Statistica 6.0 software (StatSoft, Inc., Tulsa, OK, USA).

## 3. Results

### 3.1. Composition of Yeast Produced Volatile Blends

**Figure 1:**
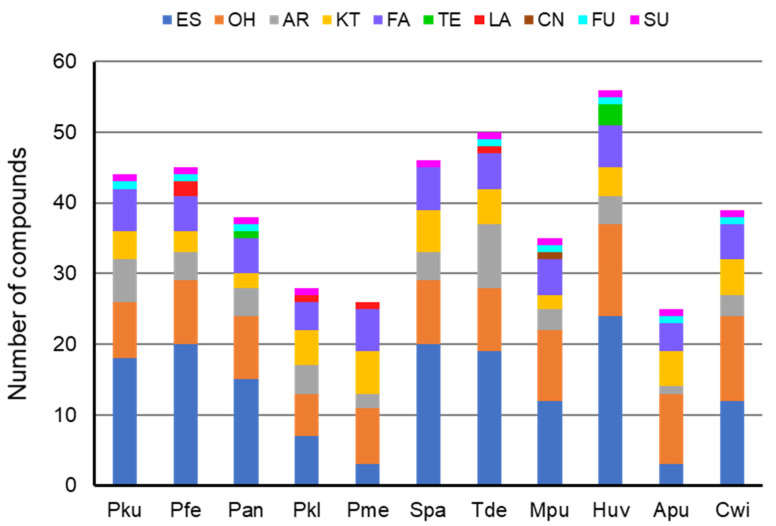
Chemical diversity of the volatile blends produced by yeasts. Pku–*Pichia kudriavzevii*, Pfe–*P. fermentans*, Pan-*P. anomala*, Pkl-*P. kluyveri*, Pme-*P. membranifaciens*, Spa-*Saccharomyces paradoxus*, Tde-*Torulaspora delbrueckii*, Mpu-*Metschnikowia pulcherrima*, Huv-*Hanseniaspora uvarum*, Apu-*Aureobasidium pullulans*, Cwi-*Cryptococcus wieringae*. Functional group of volatiles: ES–ester; OH–alcohol; AR–aromatic; KT–ketone; FA–fatty acid; TE–terpenoid; LA–lactone; CN–isothiocyanoate; FU—furane; SU–sulphide.

Analysis of yeast produced volatiles revealed 82 compounds that were exclusively present in the headspace of eleven yeast species (Table A1) or occurred at significantly larger amounts compared to those of the control samples. The esters represented by 41 compounds were accounted as the most abounded group of volatiles released by yeasts followed by 18 alcohols, nine compounds bearing aromatic moiety, eight ketones, six fatty acids, four terpenoids, three lactones, and one each of isothiocyanate, furane, and sulphide functional group was detected once (Table 1). Compounds bearing ester, alcohol, aromatic, ketone, and fatty acid moiety were detected in the volatile blends of all yeast species while terpenoid, lactone, isothiocyanoate, furane, and sulphide type volatiles were emitted by the yeast of single or few species (Figure 1).

**Figure 2:**
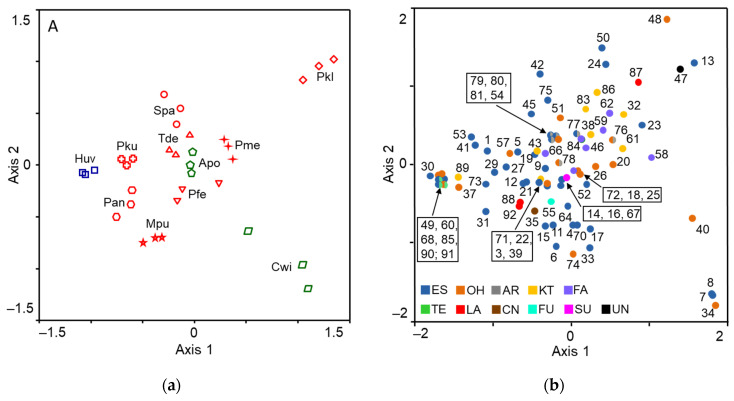
Multidimensional scaling plots: (**a**) eleven yeast species (each represented by three different isolates); (**b**) volatile compounds produced by yeasts. Volatiles were sampled from the headspace by SPME. Pku—*Pichia kudriavzevii*, Pfe—*P. fermentans*, Pan—*P. anomala*, Pkl—*P. kluyveri*, Pme—*P. membranifaciens*, Spa—*Saccharomyces paradoxus*, Tde—*Torulaspora delbrueckii*, Mpu—*Metschnikowia pulcherrima*, Huv—*Hanseniaspora uvarum*, Apu—*Aureobasidium pullulans*, Cwi—*Cryptococcus wieringae*. Apu and Cwi yeasts are more common on unripe fruits and their symbols are coloured green, Huv yeasts are the most common on medium-ripe and ripe fruits and are indicated by the blue colour, and the red colour represents yeasts, the most common on ripe fruits. The name of volatiles indicated by numbers are listed in Table 1. Functional group of volatiles: ES—ester; OH—alcohol; AR—aromatic; KT—ketone; FA—fatty acid; TE—terpenoid; LA—lactone; CN—isothiocyanoate; FU—furane; SU—sulphide; UN—unidentified.

Nonmetric multidimensional scaling analysis showed that volatile blends of all eleven species grouped in a species-specific manner (Figure 2).

**Figure 3:**
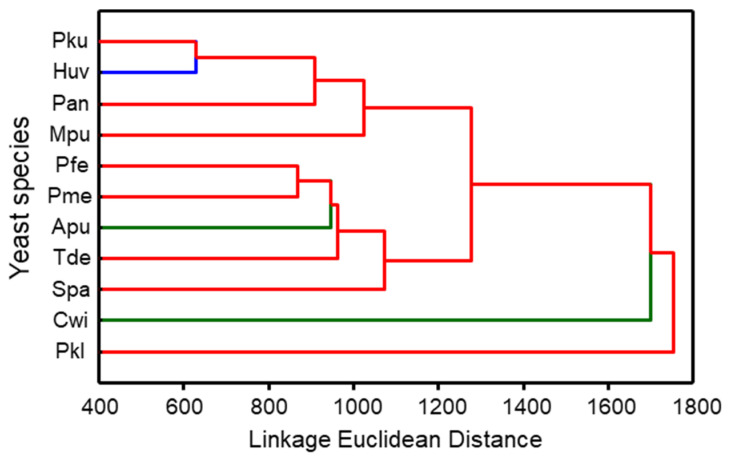
Dendrogram of odour blends sampled by SPME from the headspace of eleven yeast species. The dendrogram was obtained by cluster analysis based on Euclidean distance. The clustering was carried out based on the average of quantified volatile compounds from three different isolates per species. The different clusters were identified by visually evaluating the clustering. Pku—*Pichia kudriavzevii*, Pfe—*P. fermentans*, Pan—*P. anomala*, Pkl—*P. kluyveri*, Pme—*P. membranifaciens*, Spa—*Saccharomyces paradoxus*, Tde—*Torulaspora delbrueckii*, Mpu—*Metschnikowia pulcherrima*, Huv—*Hanseniaspora uvarum*, Apu—*Aureobasidium pullulans*, Cwi—*Cryptococcus wieringae*. Apu and Cwi yeasts are more common on unripe fruits and are represented by the green colour, Huv yeasts are the most common on medium-ripe and ripe fruits and are indicated by the blue colour, and the red colour represents yeasts, the most common on ripe fruits.

*P. kudriavzevii* and *H. uvarum* yeasts released the most similar volatile blends and together with *P. anomala* as well as *M. pulcherrima* yeasts formed a distinct cluster. Yeasts of *P. fermentans*, *P. membranifaciens, A. pullulans*, *T. delbrueckii*, and *S. paradoxus* clustered in another group. The most different blends were produced by *P. kluyveri* and *C. wieringae* yeasts (Figure 3).

### 3.2. EAD Active Compounds

**Figure 4:**
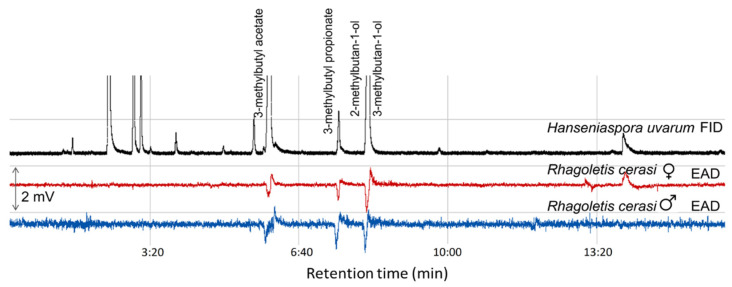
GC-EAD response of male and female *Rhagoletis cerasi* to headspace volatiles of *Hanseniaspora uvarum* yeast. FID, flame ionisation detector; EAD, electroantennographic detector; DB-Wax capillary column (30 m × 0.25 mm × 0.25 µm; Agilent Technologies, Santa Clara, CA, USA.)

GC–EAD analyses of the headspace collections from five yeast species representing three fruit ripening stages showed that antennae of *R. cerasi* flies, a pest of cherry fruits, responded to 3-methybutyl acetate, 3-methylbutyl propionate, 2-methyl-1-butanol, and 3-methyl-1-butanol (Figure 4, Table 2). Antennae of both *R. cerasi* sexes responded to all four EAD active compounds (Table 2).

Unripe fruits associated with yeast-like fungi, *A. pullulans,* and *C. wieringae* yeasts produced two alcohols, 2-methyl-1-butanol and 3-methyl-1-butanol, which elicited antennae responses of *R. cerasi* flies. *H. uvarum*, the most common yeast species inhabiting medium-ripe fruits as well *P. kudriavzevii* attributed to microbiota of ripe fruits in addition to two EAD active alcohols produced two esters, 3-methybutyl acetate and 3-methylbutyl propionate, which evoked antennae responses. Ripe fruits associated yeasts, *M. pulcherrima* released three EAD active volatiles 3-methybutyl acetate, 2-methyl-1-butanol, and 3-methyl-1-butanol (Table 2).

### 3.3. EAG Dose–Response

**Figure 5:**
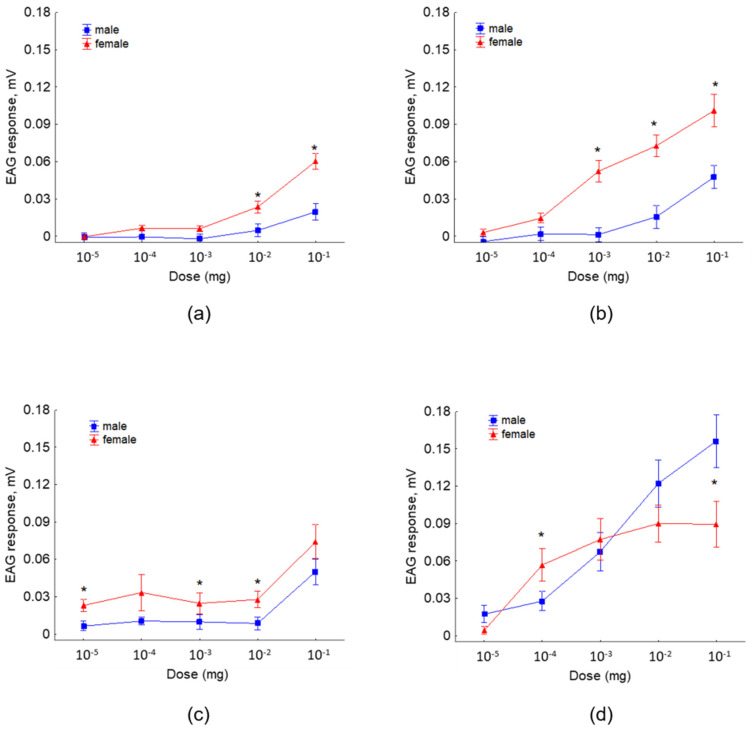
EAG responses (mean amplitude ± standard error (SE), mV) of *R. cerasi* male and female antennae to different doses (10^−5^ to 10^−1^ mg) of synthetic: (**a**) 3-methylbutyl acetate, (**b**) 3-methylbutyl propionate, (**c**) 2-methyl-1-butanol, and (**d**) 3-methyl-1-butanol. The asterisk denotes significant differences in EAG responses between sexes (paired *t* test, *p* < 0.05); each EAD test was replicated 13 times and each antenna used was from a different fly.

The antennographic responses of females to 3-methybutyl acetate and 3-methylbutyl propionate at the moderate and the higher doses were of significantly higher amplitudes compared to the responses of males (Figure 5a,b). Females showed a higher sensitivity to 2-methyl-1-butanol than males at all doses tested, and significant differences were revealed at the doses 10^−5^, 10^−3^, and 10^−2^ mg (Figure 5c). Significant stronger responses of females than males were recorded to 3-methyl-1-butanol at the dose 10^−4^ mg, while antennae stimulation with the higher doses revealed stronger responses of males compared to the responses of females (Figure 5d).

### 3.4. Behavioural Tests in Olfactometer

**Figure 6:**
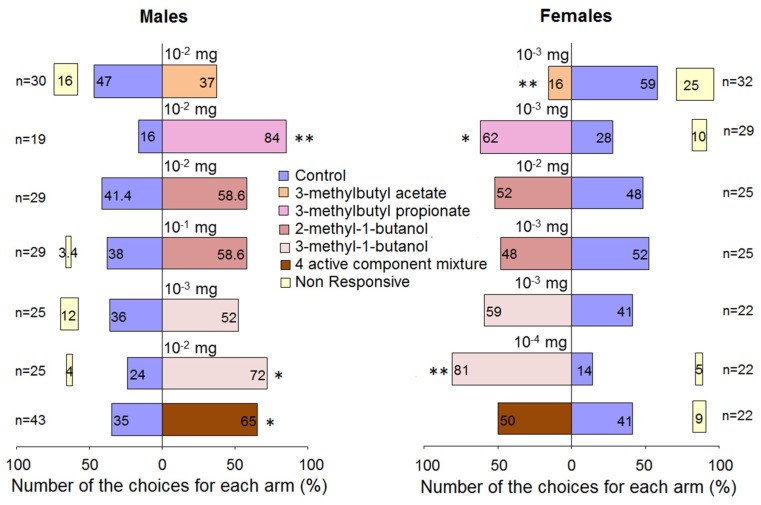
Behavioural responses of *Rhagoletis cerasi* flies in Y-tube olfactometer to EAD active volatiles. The mixture consisted of 3-methylbutyl acetate 0.55 mg, 3-methylbutyl propionate 0.05 mg, 2-methyl-1-butanol 0.012 mg, and 3-methyl-1-butanol 0.28 mg. n–number of flies tested. Stars denote significant differences in behavioural responses of flies between stimulus and control: * *p* < 0.05 and ** *p* < 0.01 by χ^2^ test.

In the two-choice tests, *R. cerasi* males showed no preference to 3-methylbutyl acetate at the dose 10^−2^ mg, while females significantly avoided the olfactometer arm bearing 3-methylbutyl acetate at the dose 10^−3^ mg. Fruit flies of both sexes significantly preferred 3-methylbutyl propionate provided at the dose of 10^−2^ mg for males and at the dose 10^−3^ mg for females (Figure 6). Flies of both sexes do not discriminate between olfactory arms bearing 2-methyl-1-butanol. Males chose 3-methyl-1-butanol at the dose 10^−2^ mg and showed no preference to a 10 times lower dose. Females preferred the alcohol at the dose of 10^−4^ mg, while a 10 times higher dose obliterated the preference.

## 4. Discussion

Yeasts produce a wide range of volatile compounds [19]. Common volatiles at large amounts emitted by different yeast species originate from primary metabolism pathways and these compounds are considered as side products or waste compounds [20]. Ethanol and ethyl acetate are the best-known examples of such volatile metabolites. Our analysis showed that yeasts of *S. paradoxus* species released high amounts of ethanol while *P. anomala*, *P. kudriavzevii*, *H. uvarum*, and *M. pulcherrima* yeasts produced the largest amounts of ethyl acetate, an ester derived from ethanol. Ethanol or ethyl acetate were the major components in the volatile bouquets of these yeast species. Interestingly, we did not detect ethanol and ethyl acetate in the headspaces of *P. kluyveri* and *P. membranifaciens* yeasts. More structurally diverse volatiles released by yeasts are derived from amino or fatty acid synthesis and degradation as well as from terpene biosynthetic pathways [19,20]. Analysis of volatile profiles of eleven yeast species isolated from sweet and sour cherry fruits showed that esters and alcohols were the most numerous groups of volatiles.

The multidimensional scaling analysis revealed that volatile blends of all yeast species were clearly separated from each other, therefore, the amount and composition of volatiles characterised yeast species. Our results are consistent with previous findings that volatile profiles of microorganisms are species-specific, reflecting specific metabolic activities of the particular microorganisms [19]. 

The ecological role of microbial volatiles falls in two major groups: they are important as semiochemicals mediating information flow at intra- and inter-specific levels; and function as promoters or inhibitors of microbial growth [21,22,23]. Yeast volatiles play an essential role in distribution of yeast from one habitat to another by attracting vector insects. Our data showed that 3-methybutyl acetate, 3-methylbutyl propionate, 2-methyl-1-butanol, and 3-methyl-1-butanol emitted by yeasts populating *P. avium* and *P. cerasus* fruits elicited electroantennographic responses in both sexes of *R. cerasi* fruit flies, a common pest of cherry fruits. Recently, it was reported that ten volatiles emitted by *P. kudriavzevii* yeast species including 3-methybutyl acetate, 3-methylbutyl propionate, and 3-methyl-1-butanolelicited antennal responses in males and females of closely related *R. batava* flies while 2-methyl-1-butanol was not EAD active [24]. 

Females showed the higher sensitivity to 3-methybutyl acetate, 3-methylbutyl propionate, and 2-methyl-1-butanol than males and the more pronounced difference in antennal sensitivity was recorded at higher doses of both esters. As far as we know, higher antennae sensitivity of females to microbial volatiles has not been reported in other tephritid fruit flies.

Two EAD active compounds 3-methylbutyl propionate and 3-methyl-1-butanol stimulated both sexes of *R. cerasi* flies to choose the olfactometer arm bearing these volatiles versus control without stimulus, while 2-methyl-1-butanol did not significantly affect the choice of flies. 3-Methyl-1-butanol is the end product of degradation of the amino acid leucine [20] and is found in volatile bouquets of many yeast species [20,25]. In our samplings, 3-methyl-1-butanol at various amounts was isolated from the emissions of all eleven yeast species. As an attractant, 3-methyl-1-butanol functions in five dipteran species [26] including one tephritid species [25]. Attractiveness of 3-methylbutyl propionate has not been reported for any dipteran species.

The two-choice test showed that *R. cerasi* females avoided the olfactometer hand bearing 3-methybutyl acetate at the dose 10^−2^ mg, while males did not significantly discriminate stimulus versus the control at even higher 10^−1^ mg dose. *R. cerasi* females prefer to lay eggs in cherries at the stage of colour change from green to yellow [27] (i.e., just at the beginning of ripening). At the early maturation stage of fruits, yeast-like fungi from *Aureobasidium* genus, yeasts from *Cryptococcus, Taphrina, Cladosporium,* and some other genus are more prevalent compared to yeast from *Pichia*, *Metschnikowia*, *Saccharomyces*, and *Torulaspora* genus inhabiting ripe fruits [3,7,28]. Analysis of chromatographic profiles of yeast emitted volatiles revealed that unripe fruits associated with yeast-like fungi, *Aureobasidium pullulans*, and *Cryptococcus wieringae* yeasts produced none or very low amounts of 3-methybutyl acetate, acting as a repellent to *R. cerasi* females. 3-Methybutyl acetate is a ubiquitous volatile in the odour bouquets of ripe and fermenting fruit-yeast complexes [29], which is in agreement with our data showing that fermenting yeasts emitted large amounts of this acetate. Contrary to *R. cerasi*, many *Drosophila* species oviposit in ripe fruits and berries and 3-methybutyl acetate released by ripe fruits associated yeasts functions as an attractant [29,30].

Ammonium acetate is reported as the most efficient food attractant for *R. cerasi* flies [31]. The number of yeast-based commercially available attractive lures such as brewer’s yeast waste, baker’s yeast (*Saccharomyces cerevisiae*), and Torula yeast (*Candida utilis*) have been used in tephritid pest control programs [32], however, none of the formulations tested showed a potential to control *R. cerasi* fruit flies. A possible explanation is that all lures released high amounts of 3-methybutyl acetate, the repellent to *R. cerasi* fruit flies. Our data suggest that yeast species selected for an efficient lure to target *R. cerasi* pests should release high amounts of 3-methylbutyl propionate and/or 3-methyl-1-butanol and do not emit 3-methybutyl acetate. Moreover, our data provide a background for the application of behaviour modifying semiochemicals in push-pull and other integrated pest management techniques to control *R. cerasi* fruit flies.

## 5. Conclusions

The odour blends emitted by yeasts were species-specific. 3-Methybutyl acetate, 3-methylbutyl propionate, 3-methylbutanol, and 2-methyl-1-butanol released by yeasts populating *P. avium* and *P. cerasus* fruits elicited electroantennographic responses and modulated behaviour of *R. cerasi* fruit flies, a common pest of cherry fruits. Therefore, these olfactory and behaviourally active compounds show potential for use in integrated pest management techniques to control *R. cerasi* fruit flies.

## Figures and Tables

**Table 1:** Odour blends of eleven yeast species and controls sampled from a headspace by the SPME technique.

No	Compound	CAS No ^3^	RI ^4^	GR ^5^	Control	*A. pullulans*	*C. wieringae*	*H. uvarum*	*P. kudriavzevii*	*P. fermentans*	*P. anomala*	*P. kluyveri*	*P. membranifac*	*S. paradoxus*	*T. delbrueckii*	*M. pulcherrima*
1	Ethyl acetate	141-78-6	>900	ES ^6^	11 ^19^ ± 6	0	0	2256 ± 657	2092 ± 150	1.6 ± 1.2	2816 ± 116	0	0	118 ± 76	202 ± 55	1586 ± 765
2	3-Methylbutanal * ^1^	590-86-3	927	AL ^7^	38 ± 8	0	0	0	0	0	0	0	0	15 ± 8	0	0
3	Ethanol	64-17-5	944	OH ^8^	0	707 ± 106	48 ± 11	386 ± 34	425 ± 5	152 ± 23	39 ± 23	0	0	2594 ± 1683	468 ± 19	677 ± 44
4	Ethyl propionate	1105-37-3	953	ES	0	0	0.9 ± 0.3	139 ± 38	149 ± 9	0	147 ± 14	0	0	1.00 ± 0.05	63 ± 8	65 ± 25
5	Ethyl 2-methylpropionate	97-62-1	960	ES	0	0	0	0	0	0.9 ± 0.7	0	0	0	0	0	0
6	Propyl acetate	109-60-4	969	ES	0	0	0	8.0 ± 0.7	0	0	99 ± 11	0	0	0	0	6.3 ± 3.6
7	Methyl 2-methylbutanoate	868-57-5	989	ES	0	0	0.8 ± 0.2	0	0	0	0	0	0	0	0	0
8	Methyl 3-methylbutanoate	556-24-1	996	ES	0	0	0.7 ± 0.2	0	0	0	0	0	0	0	0	0
9	2-Methylprop-1-yl acetate	110-19-0	994	ES	0	0	0	15 ± 5	91 ± 6	0	59 ± 19	0.4 ± 0.4	0	5.4± 2.0	12 ± 3	8.9 ± 2.1
10	Toluene *	108-88-3	1013	AR ^9^	6.3 ± 1.0	1.9 ± 0.1	1.7 ± 0.2	1.3 ± 0.6	0.8 ± 0.3	2.2 ± 0.3	0.5 ± 0.1	1.8 ± 0.8	3.8 ± 0.2	3.8 ± 1.7 ns	2.2 ± 0.7	1.6 ± 0.1
11	Ethyl butanoate	105-54-4	1015	ES	0	0	0	0.8 ± 0.5	1.1 ± 0.1	0.5 ± 0.4	0.6 ± 0.1	0	0	8.8 ± 0.8	1.2 ± 0.3	0.5 ± 0.4
12	Ethyl 2-methylbutanoate	7452-79-1	1035	ES	0	3.6 ± 0.5	1.6 ± 0.3	1.3 ± 0.5	1.2 ± 0.2	4.3 ± 3.7	3.8 ± 0.9	0	0	6.6 ± 0.2	2.6 ± 0.2	3.5 ± 0.5
13	3-Methylbutyl formate	110-45-2	1053	ES	0	0	0	0	0	0	0	1.0 ± 0.4	0	0	0	0
14	Ethyl 3-methylbutanoate	108-64-5	1053	ES	0	0.4 ± 0.1	0.10 ± 0.02	0	0	3.9 ± 2.8	0	0	0	1.7 ± 0.4	0.7 ± 0.1	0.6 ± 0.2
15	Butyl acetate	123-86-4	1055	ES	0	0	0	12 ± 2	1.8 ± 0.2	0	46 ± 4	0	0	0	0.7 ± 0.4	0.15 ± 0.03
16	2-Methylpropyl propionate	540-42-1	1064	ES	0	0	0.4 ± 0.1	1.1 ± 0.2	3.7 ± 0.5	1.7 ± 0.9	3.6 ± 0.8	496 ± 496	0	0.8 ± 0.1	3.5 ± 0.4	0.4 ± 0.2
17	2-Methylpropyl 2-methylpropionate	97-85-8	1074	ES	0	0	0.2 ± 0.1	0	0	0.3 ± 0.1	0	0	0	0	0	0
18	2-Methylpropanol	78-83-1	1093	OH	0	114 ± 6	143 ± 12	53 ± 9	61 ± 7	106 ± 17	25 ± 1	56 ± 14	26 ± 03	97 ± 6	56 ± 06	102 ± 20
19	3-Methylbutyl acetate	123-92-2	1109	ES	5.4 ± 0.2	0	4.3 ± 1.1 ns	658 ± 88	1202 ± 167	117 ± 84	1934 ± 948	70 ± 50	0	188 ± 55	623 ± 252	49 ± 19
20	1-Butanol *	71-36-3	1144	OH	0.8 ± 0.03	2.9 ± 0.6	18 ± 5	4.4 ± 0.6	0.3 ± 0.2	0.4 ± 0.4 ns	1.6 ± 0.6	0	0	2.2 ± 0.3	0.4 ± 0.2 ns	4.5 ± 1.0
21	2-Methylpropyl 2-methylbutanoate	2445-67-2	1166	ES	0	0	0	0	0	0.7 ± 0.1	0	0	0	0	0	0
22	2-Heptanone	110-43-0	1171	KT ^10^	0	10 ± 8	8.0 ± 1.7	0.5 ± 0.1	0	2.6 ± 1.1	0.2 ± 0.04	1.9 ± 0.9	3.3 ± 0.6	2.6 ± 0.6	4.8 ± 0.6	0.4 ± 0.4
23	3-Methylbutyl propionate	105-68-0	1178	ES	0.4 ± 0.02	0	0.3 ± 0.2 ns	84 ± 24	23 ± 4	20 ± 18	71 ± 8	2937 ± 1962	1.6 ± 0.7	11 ± 1	98 ± 11	1.4 ± 0.5
24	3-Methylbutyl 2-methylpropionate	2050-01-3	1184	ES	0	0	0	0	0	4.5 ± 1.8	0	0	0.5 ± 0.2	0.3 ± 0.1	12 ± 3	0
25	2-Methyl-1-butanol	137-32-6	1205	OH	0	80 ± 11	262 ± 64	129 ± 25	201 ± 61	209 ± 19	96 ± 11	154 ± 15	29 ± 4	172 ± 53	199 ± 37	193 ± 4
26	3-Methyl-1-butanol	123-51-3	1212	OH	0.8 ± 0.1	640 ± 86	889 ± 297	461 ± 84	874 ± 74	1876 ± 117	416 ± 21	568 ± 118	687 ± 169	1418 ± 93	1787 ± 61	644 ± 226
27	Ethyl hexanoate	123-66-0	1225	ES	0	0.8 ± 0.4	0	0.6 ± 0.1	0.7 ± 0.2	3.6 ± 3.6	0	0	0	16 ± 9	0.1 ± 0.03	0
28	Styrene *	100-42-5	1237	AR	11 ± 4	2.4 ± 2.0	19 ± 5 ns	0.5 ± 0.2	3.5 ± 2.9 ns ^20^	5.2 ± 2.6 ns	19 ± 24 ns	14 ± 0.7 ns	7.2 ± 2.1 ns	9 ± 2 ns	10 ± 3 ns	11 ± 6 ns
29	3-Methylbutyl butanoate	106-27-4	1256	ES	0	0	0	0.4 ± 0.1	0	0.5 ± 0.02	0	0	0	0	0.4 ± 0.1	0
30	Hexyl acetate	142-92-7	1265	ES	0	0	0	0.45 ± 0.03	0	0	0	0	0	0	0	0
31	3-Methylbutyl 2-methylbutanoate	27625-35-0	1272	ES	0	0	0	0	0	3.0 ± 0.8	1.5 ± 0.3	0	0	0	0	0
32	2-hydroxy-3-butanone	513-86-0	1274	KT	0	2.2 ± 0.9	3.6 ± 0.4	10 ± 1	0	0	0	0.7 ± 0.1	0	6.7±0.7	2.2 ± 0.2	17 ± 2
33	3-Methylbutyl 3-methylbutanoate	659-70-1	1288	ES	0	0	0.4 ± 0.2	0	0	8.9 ± 3.9	0.7 ± 0.1	0	0	0	0	0
34	2-Methylpentanol	105-30-6	1299	OH	0	0	13 ± 3	0	0	0	0	0	0	0	0	0
35	2-Methylpropyl isothiocyanoate	591-82-2	1304	CN ^11^	0	0	0	0	0	0	0	0	0	0	0	0.4 ± 0.1
36	2,5- Dimethyl pyrazine *	123-32-0	1314	PY ^12^	9.1 ± 0.5	1.2 ± 0.2	22 ± 13 ns	3.8 ± 0.6	3.4 ± 0.4	4.8 ± 0.2	2.0 ± 0.6	6.0 ± 3.1 ns	7.6 ± 0.4 ns	6.4±0.5 ns	3.9 ± 0.5	4.6 ±0.6
37	2-Heptanol	543-49-7	1321	OH	0	2.0 ± 1.3	0	0.50 ± 0.01	0	0	0	0	0	0	0	0.9 ± 0.7
38	6-Methyl 5-hepten-2-one	110-93-0	1327	KT	0.4 ± 0.1	4.4 ± 3.8	103 ± 23	0.29 ± 0.03 ns	0	0.7 ± 0.1	0	0.8 ± 0.5 ns	2.8 ± 1.0	1.1 ± 0.2	0.5 ± 0.4 ns	0
39	Ethyl heptanoate	106-30-9	1331	ES	0	0	0	0	0.4 ± 0.1	0	0	0	0	0	0	0
40	1-Hexanol	111-27-3	1346	OH	0	0	0.5 ± 0.2	0	0	0	0	0	4.3 ± 1.9	0	0	0
41	Heptyl acetate	112-06-1	1369	ES	0	0	0	0.7 ± 0.1	0	0	0	0	0	0.3 ± 0.2	0	0
42	2-Ethylhexyl acetate	103-09-3	1382	ES	0	0	0	0	0	0	0	0	0	1.0 ± 0.4	0	0
43	Nonan-2-one	821-55-6	1383	KT	0	49 ± 28	13 ± 4	0.5 ± 0.2	0.3 ± 0.1	2.3 ± 1.0	1.7 ± 0.4	1.3 ± 0.2	2.9 ± 1.0	5.2 ± 4.2	2.8 ± 0.6	0
44	2,3,5-Trimethyl pyrazine *	14667-55-1	1397	PY	0.6 ± 0.1	0.6 ± 0.1 ns	0.8 ± 0.1 ns	0.58 ± 0.01 ns	0.6 ± 0.04 ns	0.6 ± 0.1 ns	0.6 ± 0.1 ns	0.4 ± 0.2 ns	0.7 ± 0.2 ns	0.8 ± 0.1 ns	0.5 ± 0.4 ns	0.60 ± 0.02 ns
45	Ethyl octanoate	106-32-1	1427	ES	0	0	0	4.1 ± 0.1	3.7 ± 0.6	0.2 ± 0.2	0	0	0	5.6 ± 5.2	0	0
46	Acetic acid	64-19-7	1439	FA ^13^	0.3 ± 0.01	0.7 ± 0.01	4.5 ± 3.5 ns	69 ± 11	5.3 ± 3.9 ns	0.6 ± 0.1	7.9 ± 3.0	0	0.8 ± 0.1	0.79 ± 0.04	0.77 ± 0.04	1.1 ±0.4
47	Unknown		1440		0	0	0	0	0	0	0	10 ± 4	0	0	0	0
48	1-Octen-3-ol	3391-86-4	1450	OH	0	0	0	0	0	0	0	0	0.12 ± 0.01	0	0	0
49	1-Heptanol	111-70-6	1454	OH	0	0	0	0.8 ± 0.2	0	0	0	0	0	0	0	0
50	3-Methylbutyl hexanoate	2198-61-0	1457	ES	0	0	0	0	0	0	0	0	0.25 ± 0.01	0.8 ± 0.2	0	0
51	6-Methyl-5-hepten-2-ol	1569-60-4	1460	OH	0	1.1 ± 0.7	12 ± 3	0	0	0	0	0	0	0	0	0
52	MHMP ^2^	40348-72-9	1462	ES	0	0	0	0	0	0	0	0	0	0	1.2 ± 0.3	0
53	Octyl acetate	112-14-1	1472	ES	0	0	0	0.3 ± 0.1	0	0	0	0	0	1.3 ± 0.9	0	0
54	2-Ethylhexanol	104-76-7	1483	OH	0.3 ± 0.03	1.5 ± 0.1	1.8 ± 0.2	0.8 ± 0.1	0.6 ± 0.1	0.6 ± 0.1	1.3 ± 0.4	0.8 ± 0.4 ns	1.7 ± 0.8	0.9 ± 0.1	1.1 ± 0.2	0.71 ± 0.03
55	Acetylfuran	1192-62-7	1496	FU ^14^	0	0.3 ± 0.1	0.3 ± 0.2	0.6 ± 0.1	0.2 ± 0.1	0.6 ± 0.3	0.7 ± 0.3	0	0	0	0.5 ± 0.1	0.19 ± 0.02
56	Benzaldehyde *	100-52-7	1505	AR, AL	9.9 ± 2.7	2.0 ± 1.6	0.4 ± 0.1	0.4 ± 0.1	0.28 ± 0.04	0.29 ± 0.04	0.18 ± 0.02	1.0 ± 0.2	0.20 ± 0.02	0.23 ± 0.01	0.20 ± 0.03	0.3 ± 0.1
57	2-Nonanol	628-99-9	1524	OH	0	0.05 ± 0.04	0	1.1 ± 0.2	0	0.2 ± 0.1	0	0	0	0.3 ± 0.1	0.4 ± 0.1	0.5 ± 0.1
58	Propionic acid	79-09-4	1526	FA	0.3 ± 0.04	0	2.9 ± 2.0 ns	6.7 ± 1.7	2.2 ± 0.1	0.7 ± 0.5 ns	1.0 ± 0.4	0.9 ± 0.2	1.6 ± 0.3	2.3 ± 0.4	2.4 ± 0.5	0.3 ± 0.1 ns
59	2-Methylpropionic acid	79-31-2	1557	FA	4.9 ± 0.5	0	12 ± 6 ns	34 ± 5	26 ± 2	13 ± 7	9.9 ± 2.2	8.3 ± 1.6	20 ± 3	1.8 ± 1.2 ns	1.9 ± 0.8 ns	1.9 ± 1.0 ns
60	2-Decanol	1120-06-5	1574	OH	0	0	0	0.7 ± 0.2	0	0	0	0	0	0	0	0
61	2-Undecanone	112-12-9	1594	KT	0	1.1 ± 0.6	0.6 ± 0.1	0	0	0	0	0.2 ± 0.1	1.1 ± 0.2	0.20 ± 0.02	0.8 ± 0.2	0
62	Butanoic acid	107-92-6	1614	FA	1.0 ± 0.1	0.2 ± 0.04	2.6 ± 1.8 ns	8.5 ± 1.7	2.7 ± 0.1	0.7 ± 0.5 ns	9.0 ± 2.1	0.3 ± 0.2	1.1 ± 0.1 ns	0.2 ± 0.1	0.21 ± 0.04	0.3 ± 0.2
63	Acetophenone *	98-86-2	1625	AR, KT	1.8 ± 0.2	0.7 ± 0.1	0.7 ± 0.1	1.1 ± 0.1	0.8 ± 0.04	0.4 ± 0.4	0.9 ± 0.1	1.1 ± 0.8 ns	1.3 ± 0.1 ns	1.6 ± 1.0 ns	1.5 ± 0.1 ns	0.6 ± 0.1
64	Ethyl decanoate	110-38-3	1632	ES	0	0	0	4.0 ± 1.2	0.9 ± 0.1	0.5 ± 0.5	0.2 ± 0.1	0	0	2.3 ± 2.2	0	0
65	2-Furanmethanol *	98-00-0	1649	OH	1.8 ± 0.1	1.7 ± 1.5 ns	1.2 ± 0.4 ns	0	0	0	0	0	0	0	1.7 ± 0.4	0
66	3-Methylbutanoic acid	503-74-2	1658	FA	20 ± 2	0.26 ± 0.02	58 ± 39	261 ± 66	136 ± 9	37 ± 25 ns	44 ± 12	21 ± 6 ns	71 ± 14	6.8 ± 5.6	4.5 ± 2.9	4.6 ± 4.1
67	3-Hydroxypropyl methylsulphide	505-10-2	1700	SU ^15^	0	0.18 ± 0.03	0.4 ± 0.1	0.33 ± 0.04	2.0 ± 1.0	1.3 ± 0.7	6.6 ± 1.1	0.9 ± 0.4	0	0.8 ± 0.2	7.5 ± 2.2	0.5 ± 0.2
68	Geranyl acetate	16409-44-2	1764	TE ^16^, ES	0	0	0	0.7 ± 0.2	0	0	0	0	0	0	0	0
69	Methoxy-phenyl-oxime *	67160-14-9	1768	IM ^17^	3.1 ± 0.7	1.2 ± 0.2	2.9 ± 0.4 ns	0.6 ± 0.3	1.3 ± 0.1	0.7 ± 0.2	0.43 ± 0.02	1.7 ± 0.4 ns	1.0 ± 0.3	0.6 ± 0.2	0.6 ± 0.2	1.4 ± 0.4
70	Ethyl 2-phenylacetate	119-43-7	1767	AR, ES	0	0	0	0	0.6 ± 0.2	0	0.3 ± 0.1	0	0	0	0	0
71	2-Phenylethyl acetate	103-45-7	1794	AR, ES	0	0	0	148 ± 13	353 ± 48	2.1 ± 1.6	88 ± 8	7.8 ± 6.3	0	8.0 ± 3.4	65 ± 17	11 ± 8
72	Hexanoic acid	122-70-3	1816	AR, ES	0	0	0.8 ± 0.1	0	0.4 ± 0.1	0.3 ± 0.2	0	0	0	0.6 ± 0.1	0	0
73	Ethyl dodecanoate	106-33-2	1823	ES	0	0	0	1.7 ± 1.0	0.1 ± 0.03	0.3 ± 0.1	0	0	0	0	0	0
74	Geraniol	106-24-1	1842	TEOH	0	0	0.5 ± 0.1	0	0	0	0.5 ± 0.1	0	0	0	0	0
75	3-Methylbutyl decanoate	2306-91-4	1843	ES	0	0	0	0	0	0	0	0	0	0.2 ± 0.1	0	0
76	Phenyl methanol	100-51-6	1854	AR, OH	0.7 ± 0.04	0	0.7 ± 0.2 ns	5.1 ± 0.9	2.9 ± 0.5	1.8 ± 1.0 ns	1.2 ± 0.3 ns	1.2 ± 0.2	0.6 ± 0.2 ns	0.7 ± 0.2 ns	1.1 ± 0.2 ns	2.2 ± 0.3
77	2-Phenylethyl propionate	122-70-3	1862	AR, ES	0	0	0	18 ± 4	6.3 ± 0.7	0	1.1	1.1 ± 0.9	0	0	172 ± 38	0
78	2-Phenyl ethanol	60-12-8	1890	AR, OH	0.2 ± 0.04	362 ± 102	20 ± 2	336 ± 46	567 ± 19	865 ± 206	235 ± 17	432 ± 57	814 ± 17	271 ± 83	448 ± 87	490 ± 320
79	2-Phenylethyl butanoate	103-52-6	1949	AR, ES	0	0	0	0	0	0	0	0	0	0	0.2 ± 0.1	0
80	2-Phenylethyl 2-methylbutanoate	24817-51-4	1955	AR, ES	0	0	0	0	0	0	0	0	0	0	4.3 ± 1.8	0
81	2-Phenylethyl 3-methylbutanoate	140-26-1	1973	AR, ES	0	0	0	0	0	0	0	0	0	0	0.11 ± 0.02	0
82	Phenol *	87-66-1	1979	AR, OH	0.4 ± 0.04	0.38 ± 0.01 ns	0.5 ± 0.1 ns	0.15 ± 0.01	0.07 ± 0.03	0.4 ± 0.1 ns	0.5 ± 0.1 ns	0.2 ± 0.1 ns	0.6 ± 0.2 ns	0.49 ± 0.02	0.3 ± 0.1 ns	0.30 ± 0.04 ns
83	Pentadecan-2-one	2345-28-0	2010	KT	0	0	0	0	0.2 ± 0.1	0	0	0	1.2 ± 0.3	0.08 ± 0.06	0	0
84	Octanoic acid	124-07-2	2065	FA	0.3 ± 0.02	1.0 ± 0.2	0	0.3 ± 0.1 ns	0.6 ± 0.1	0	0	0	0.27 ± 0.03 ns	0.20 ± 0.01	0	0
85	3-Methylbutyl dodecanoate	6309-51-9	2089	ES	0	0	0	2.3 ± 0.4	0	0	0	0	0	0	0	0
86	Hexadecan-2-one	18787-63-8	2118	KT	0	0	0	0	0.4 ± 0.1	0	0	0	0.6 ± 0.2	0	0	0
87	gamma-Decalactone	706-14-9	2125	LA ^18^	0	0	0	0	0	0	0	1.2 ± 0.2	5.2 ± 1.2	0	1.3 ± 0.8	0
88	6-Pentyl-5,6 dihydro-2H-pyran-2-one	54814-64-1	2222	LA	0	0	0	0	0	2.3 ± 1.1	0	0	0	0	0	0
89	Heptadecan-2-one	2922-51-2	2230	KT	0	0	0	0	2.1 ± 0.6	0	0	0	0	0	0	0
90	Farnesyl acetate	4128-17-0	2247	TE, ES	0	0	0	1.1 ± 0.2	0	0	0	0	0	0	0	0
91	Farnesol	4602-84-0	2287	TE, OH	0	0	0	1.7 ± 0.3	0	0	0	0	0	0	0	0
92	4-Hydroxy-6-pentyloxan-2-one	36555-25-6	2456	LA	0	0	0	0	0	1.2 ± 0.4	0	0	0	0	0	0

^1^ Compounds indicated by star mark were excluded from multivariate analysis, ^2^ MHMP-methyl 2-hydroxy-4-methylpentanoate, ^3^ CAS No–chemical abstract service number, ^4^ RI–retention index (DB-Wax fused silica capillary column 30 m × 0.25 mm i.d., 0.25 µm film thickness), ^5^ GR—group of a chemical compound, ^6^ ES—ester, ^7^ AL—aldehyde, ^8^ OH—alcohol, ^9^ KT—ketone, ^10^ AR—aromatic, ^11^ CN—isothiocyanoate, ^12^ PY—pyrazine, ^13^ FA—fatty acid, ^14^ FU—furane, ^15^ sulphide, ^16^ TE—terpenoid, ^17^ IM—imine, ^18^ LA—lactone, ^19^ mean ± standard error of the mean (means are the absolute amounts expressed as areas under the chromatographic peaks and have to be read as numbers times 1000), ^20^ ns—not significantly different compare to control (Nonparametric Mann–Whitney U test, *p* < 0.05), three different isolates of each yeast species have been used; control samples were obtained by collecting background volatiles from YPD-agar plates without yeast.

**Table 2:** Electroantennographic responses of *R. cerasi* flies to volatiles present in the headspace of five yeast species.

Yeast Species	3-MBA ^1^		3-MBP ^2^		2-MBOH ^3^		3-MBOH ^4^	
	Male	Female	Male	Female	Male	Female	Male	Female
*Aureobasidium pullulans*	- ^5^				3 (3)	3 (3)	3 (3)	3 (3)
*Cryptococcus wieringae*					4 (4)	3 (3)	4 (4)	3 (3)
*Hanseniaspora uvarum*	4 ^6^ (4) ^7^	4 (4)	4 (4)	3 (4)	4 (4)	4 (4)	4 (4)	4 (4)
*Pichia kudriavzevii*	5 (7)	4 (4)	5 (7)	4 (4)	7 (7)	4 (4)	7 (7)	4 (4)
*Metschnikowia pulcherrima*	1 (3)	3 (4)			3 (3)	4 (4)	3 (3)	4 (4)
Total	10 (14)	11 (12)	9 (11)	7 (8)	21 (21)	18 (18)	21 (21)	18 (18)

^1^ 3-Methylbutyl acetate, ^2^ 3-methylbutyl propionate, ^3^ 2-methyl-1-butanol, ^4^ 3-methyl-1-butanol, ^5^ compound was not detected in the headspace, ^6^ Number of antennae responded, ^7^ Number of antennae tested.

## Data Availability

Not applicable.

## Appendix A

**Table A1:** List of yeast isolates analysed in this study.

Yeast Species	Strain	GenBank Reference	Identity (%)
*Aureobasidium pullulans*	PA-4-13	MN400109	100
	PC-5-28	HQ909088	99.6
	PC-4-8	MW361317	99.8
*Cryptococcus wieringae*	PC-4-6	KF981864	100
	PC-4-9	KF981864	100
	PA-5-41	KF981864	100
*Metschnikowia pulcherrima*	PC-4-5	MF574308	100
	PA-5-13	KT029787	99.7
	PA-5-47	MK352050	100
*Hanseniaspora uvarum*	PA-5-27	MF062209	100
	PC-3-8	MK352020	99.9
	PC-5-12	KY103573	100
*Pichia kudriavzevii*	PA-1-15	MF685423	100
	PC-4-25	MF685411	100
	PC-4-19.3	MG015972	99.6
*Pichia fermentans*	PC-5-47	FJ713081	100
	PC-4-19.1	MF462777	100
	PA-4-39	KY104537	100
*Pichia kluyveri*	PA-4-30	JX103190	100
	PA-4-34	KY108823	99.8
	PC-4-35	KC510043	100
*Pichia membranifaciens*	PC-3-36	JX188207	100
	PC-2-71	FJ231461	100
	PA-2-55	JX188207	99.6
*Saccharomyces paradoxus*	PA-5-14	FJ713072	100
	PC-3-33	FJ713072	100
	PC-3-59	KY105204	99.9
*Torulaspora delbrueckii*	PA-4-16	KY105641	100
	PA-5-17	MN371902	99.8
	PC-2-42	MK352012	100
*Pichia anomala*	PC-5-5	KJ527050	100
	PA-5-18	MH248067	100
	PC-5-24	MK343437	100
